# Bacteremia detected on peripheral blood smear in small animal patients presenting to the Emergency Department and its association with prognosis to discharge

**DOI:** 10.3389/fvets.2025.1550732

**Published:** 2025-06-04

**Authors:** Summer Scout Ford, Megan Whelan, Patty Ewing, Virginia B. Sinnott-Stutzman

**Affiliations:** ^1^Department of Emergency and Critical Care, Angell Animal Medical Center, Boston, MA, United States; ^2^Department of Pathology, Angell Animal Medical Center, Boston, MA, United States; ^3^BluePearl, Tampa, FL, United States

**Keywords:** bacteremia, peripheral blood smear, hyperglycemia, pathologist, emergency, sepsis

## Abstract

**Objective:**

Detection of bacteremia on peripheral blood smear (PBS) is rare and may be a poor prognostic indicator for small animal patients. This study aimed to determine the relationship between bacteremia on PBS and survival to discharge in clinically ill patients presenting through the Emergency Department (ED).

**Methods:**

This retrospective study analyzed data from two veterinary tertiary care facilities from 2014 to 2024. Records from 16 client-owned animals presenting to the ED with PBS-detected bacteremia were reviewed. The primary outcome was survival to discharge. Secondary outcomes evaluated associations between survival in these patients with glucose level, leukocyte count, toxic change, band neutrophils, total bilirubin, blood pressure, and antibiotic use. Statistical comparisons between categorical data were made using Fisher’s exact test. A *p*-value of <0.05 was considered significant.

**Results:**

In-hospital mortality of the 16 patients was 75% (12/16). Hyperglycemia was positively associated with survival (*p* = 0.0099). All survivors were cats. No other parameters showed statistical significance between survivors and non-survivors.

**Conclusion:**

PBS-detected bacteremia in clinically ill small animals was associated with a high in-hospital mortality in this study. Further investigation is warranted to better understand its clinical relevance and potential diagnostic utility.

## 1 Introduction

The primary objective of this study was to evaluate any association between bacteremia when detected on peripheral blood smear (PBS) in clinically ill small animal patients, and prognosis to discharge. Bacteremia, characterized by the presence of bacteria in the blood, is an uncommon, but concerning finding on a PBS (1, 2). Unlike conditions such as sepsis, which involves a life-threatening infection and dysregulated or insufficient host response, bacteremia may not necessarily indicate an advanced infection on its own (3, 4). Given the lower bacterial load in the blood that can be associated with bacteremia, it may be transient and handled by the immune system without medical intervention (3, 5). However, the detection of bacteremia on PBS in clinically unwell small animals raises concern for more severe illness and poorer prognosis (1, 6). Although bacterial load was not directly measured in the present study, detection of bacteremia on PBS in the emergency setting may serve as an early marker of disease severity.

There can be many different sources of bacteremia ranging from a peripheral wound, surgical site, venous access site, blood contamination, or a source of infection throughout the body leading to hematogenous spread (4). Though a PBS does not replace the recommendation for blood culture and further diagnostics (7, 8), it can serve as a rapid diagnostic tool to guide the initial treatment plan and expedite antibiotic therapy in lieu of pending or absent blood culture results (2, 9, 10). There is currently very limited veterinary literature evaluating whether the presence of bacteremia identified on PBS correlates with prognosis to discharge (2, 9).

The objective of this study was to report in-hospital mortality associated with PBS-detected bacteremia in clinically ill small animals, regardless of underlying cause. Our primary hypothesis was that the detection of bacteremia on PBS would be associated with a poor prognosis to discharge. A secondary aim was to evaluate whether survival in this bacteremic population was associated with other clinical parameters, such as blood glucose concentration (11).

## 2 Materials and methods

### 2.1 Study design

This multi-institutional, retrospective study was conducted at two veterinary tertiary care facilities. The medical record database at each facility was searched for the word “bacteremia.” Patients where bacteremia was identified on PBS by pathologist review at time of presentation or during hospitalization were considered for inclusion. Medical records spanning 2014 to 2024 were reviewed to extract relevant clinical data, including diagnostic findings and treatment outcomes. The primary outcome assessed was survival to discharge, with secondary outcomes describing the relationship between survival and various clinical parameters in this bacteremic patient population. The parameters that were evaluated included the patient’s signalment, serum glucose concentration, white blood cell count (WBC), presence of toxic change, band neutrophils, serum total bilirubin, blood pressure, and antibiotic usage while in hospital and over the 6 months prior to hospital admission.

### 2.2 Diagnostic techniques

Blood smears were prepared and stained using Wright-Giemsa stain. These smears were then examined by a board-certified clinical pathologist, with detailed descriptions of bacteria and cell morphology noted. At the included institutions, a pathologist review of a PBS is triggered by a technologist alert or CBC abnormality. Hypotension was defined as a systolic blood pressure of less than or equal to 90 mmHg. A mean arterial pressure of less than or equal to 60 mmHg was also included if an oscillometric blood pressure measurement was recorded in the record (12). The lowest blood pressure recorded during the hospitalization period was used. Antibiotic usage was recorded both during hospitalization and, when documented, within the 6 months prior to hospital admission (Table 1). Biochemical parameters were categorized using species-specific preset laboratory reference ranges, in combination with literature reference limits as detailed in Table 1 (12, 13). Blood glucose and total bilirubin concentrations were measured from serum samples. The biochemical results recorded were from initial blood work submitted at time of admission.

**Table 1:** Survival outcomes by clinical parameters.

Parameter/control	Survivors	Non-survivors	Fisher’s exact test (*p*-value)
1. Leukocytosis (>15.1 K/uL): (bearded dragons > 19 K/uL)^*^ (rabbits > 10.8 K/uL)^*^	1	4	1
1. Non-leukocytosis (<15.1 K/uL): (bearded dragons < 19 K/uL)^*^ (rabbits < 10.8 K/uL)^*^	4	8	
2. Leukopenia (<4.3 K/uL): (bearded dragons < 1.45 K/uL)^*^ (rabbits < 4.1 K/uL)^*^	2	4	1
2. Non-leukopenia (>4.3 K/uL): (bearded dragons > 1.45 K/uL)^*^ (rabbits > 4.1 K/uL)^*^	3	8	
3. **Hyperglycemia** (>126 mg/dL): (bearded dragons > 333 mg/dL)^*^ (rabbits > 161 mg/dL)^*^	4	1	**0.0099**
3. **Non-hyperglycemia** (<126 mg/dL): (bearded dragons < 333 mg/dL)^*^ (rabbits < 161 mg/dL)^*^	1	11	
4. Hypoglycemia (<70 mg/dL): (bearded dragons < 108 mg/dL)^*^ (rabbits < 109 mg/dL)^*^	0	4	0.26
4. Non-hypoglcemia (>70 mg/dL): (bearded dragons > 108 mg/dL)^*^ (rabbits > 109 mg/dL)^*^	5	8	
5. Toxic change	3	11	0.19
5. No toxic change	2	1	
6. Band neutrophils	3	10	0.54
6. No band neutrophils	2	2	
7. Hyperbilirubinemia (>0.3 mg/dL): (bearded dragons > 1.4 mg/dL)^*^ (rabbits > 0.5 mg/dL)^*^	4	8	1
7. Non-hyperbilirubinemia (<0.3 mg/dL): (bearded dragons < 1.4 mg/dL)^*^ (rabbits < 0.5 mg/dL)^*^	1	2	
8. Hypotension (MAP ≤ 60 mmHg; SAP ≤ 90 mmhg)	0	6	0.21
8. Not hypotensive (MAP > 60 mmHg; SAP > 90 mmhg)	3	5	
9. Antibiotics in last 6 months	0	3	0.53
9. No antibiotics in last 6 months	4	9	
10. Antibiotics during hospitalization	4	11	1
10. No antibiotics during hospitalization	0	0	

This table outlines the secondary outcomes related to survival at discharge for the animals where the variable was recorded in the medical record. Each row compares the survival between patients with specific clinical parameters and their controls. Significant results (*p* < 0.05) are bolded, indicating key findings that correlate with survival rates. ^*^See Carpenter and Harms (13).

For the purpose of identifying possible sepsis in this patient population, sepsis was defined for the use in this study as a combination of a positive blood culture and available criteria that may indicate organ dysfunction (4, 8). The criteria that were considered were a leukocytosis or leukopenia, presence of band neutrophils, hypotension, and hyperbilirubinemia. These criteria were extrapolated from the human sequential organ failure assessment (SOFA) score and SIRS criteria for dogs and cats (4, 8, 14).

### 2.3 Statistical analysis

Statistical comparisons were performed using Fisher’s exact test to assess associations between categorical variables and the primary outcome being survival to discharge. Due to the small sample size, subgroup analysis by species was not statistically feasible. Descriptive statistics for each species are provided, and inferential statistics were conducted on the combined dataset. Continuous clinical variables (i.e., glucose level, WBC count, total bilirubin, and blood pressure) were converted into categorical variables based on established species-specific reference intervals and thresholds derived from veterinary literature (see Section 2.2). A *p*-value of <0.05 was considered statistically significant. Descriptive statistics, including medians, interquartile ranges (IQR), and full ranges, were reported for continuous variables in Table 2, with group stratification by survival status.

**Table 2:** Medians and ranges of quantitative biochemical parameters in survivors vs. non-survivors.

Parameter	Survivors	Non-survivors			
	Cats (5)	Cats (3)	Dogs (7)	Bearded dragon (1)	Rabbit (1)
Leukocyte count (K/uL)	Median: 9.60 (IQR: 22.95)	Median: 11.46	Median: 9.30 (IQR: 21.27)	0.15	1.7
	Range: 0.11–36.5	Range: 1.76–17.6	Range: 1.05–26.83		
Glucose level (mg/dL)	Median: 171 (IQR: 76)	Median: 106	Median: 96 (IQR: 40)	73	24
	Range: 94–205	Range: 100–420	Range: 60–112		
Total bilirubin (mg/dL)	Median: 1.5 (IQR: 2.35)	Median: 1.1	Median: 0.6 (IQR: 0.4)	n/a	n/a
	Range: 0.1–3.5	Range: 0.2–1.7	Range: 0.1–8.7		

This table outlines the median, IQR, range of the quantitative biochemical data collected for this patient population, and is separated into the survivor and non-survivor groups for comparison. All survivors in this population were cats. The non-survivors have been further sub-divided into dogs, cats, and exotic species. The values for the exotic species (rabbit, bearded dragon) have been provided separately due to difference in reference ranges between species. One cat was counted to twice to represent separate data at each ED admission. IQR was only able to be calculated for patient groups > 4.

## 3 Results

Sixteen client-owned animals presenting to the ED from 2015 to 2024 were found to have bacteremia (see Supplementary Figure 1) noted on PBS at time of presentation or during hospitalization, and met the inclusion criteria for this study. The study documented high in-hospital mortality with 75% (12/16) of the animals not surviving to discharge. Euthanasia accounted for 58% (7/12) of cases that did not survive to discharge. Also of note, all surviving patients (4/16) were cats. All patients included in the study were admitted to the ED for a variety of clinical signs. Presenting complaints were often nonspecific and commonly included signs such as lethargy, vomiting, and hyporexia. None of the patients included had been previously determined to be septic, or had positive blood cultures prior to detection of bacteria on PBS (4, 14). Duration of hospitalization for non-survivors ranged from 1 to 11 days, while survivors were hospitalized for 1 to 5 days. One cat presented to the Emergency Department (ED) on two separate occasions and was found to be bacteremic on PBS at both visits. At the first presentation, the cat was treated as an outpatient and survived to discharge. However, the cat re-presented 24 h later with clinical decline, and repeated PBS again showed bacteremia. This patient was ultimately euthanized during the second hospitalization. For the purpose of clinical parameter comparison, both visits are included in the analysis (Table 1), once under “survivor” and once under “non-survivor,” to reflect the findings at each point of care. However, for primary outcome reporting (i.e., overall survival), this cat is counted only once, as a non-survivor.

The study population consisted of 7 dogs (43%), 7 cats (43%), one Holland Lop rabbit (6%), and one bearded dragon (6%). Dog breeds included German Shepherd (1), Great Pyrenees (1), Wire Fox Terrier (1), Miniature Pinscher (1), Miniature Poodle (1), Beagle (1), and an Italian Greyhound (1). Their ages ranged from 7 months to 19 years with a median age of 10.45 years. Among the cats, the Domestic Shorthair (4), Bengal (1), Siberian (1), and British Shorthair (1) were represented, with ages from 4 months to 15 years with a median age of 6.33 years. Of the patients, 71% (5/7) of the dogs and 57% (4/7) of the cats were male. Of the dogs included, 43% (3/7) were castrated males, 28.5% (2/7) were intact males, and 28.5% (2/7) were spayed females. Of the cats included, 28.5% (2/7) were castrated males, 28.5% (2/7) were intact males, and 43% (3/7) were spayed females.

Pathologist review of PBS described confirmed intracellular phagocytized bacteria within neutrophils, monocytes, or thrombocytes in 10 cases, suspected intracellular bacteria in 3 cases, and extracellular bacteria in 3 cases. Several patients exhibited both intra-and extracellular organisms. Morphological descriptions of bacteria varied (small rods, cocci, diplococci, coccobacilli), but no consistent differences were noted between survivors and non-survivors. In exotic species (rabbit and bearded dragon), heterophil rather than neutrophil toxic changes predominated.

Neither the presence of leukocytosis or leukopenia was associated with survival (*p =* 1). The presence of toxic change and band neutrophils was not significantly associated with survival (*p =* 0.19 and *p =* 0.54, respectively). Similarly, elevated bilirubin levels did not correlate significantly with survival outcomes (*p =* 1). No patients with hypotension survived (0 alive, 6 deceased), but hypotension was not associated with survival when compared with normotensive and hypertensive patients (*p* = 0.21). The use of antibiotics, both in the 6 months prior to presentation and during hospitalization, was not significantly associated with survival (*p* = 0.53 and *p* = 1, respectively). Of the 17 total ED presentations, 7 received antibiotics prior to PBS evaluation. Three of those had documentation of antibiotic administration within the 6 months preceding presentation. Antibiotics administered during hospitalization included ampicillin-sulbactam (15/17), enrofloxacin (10/17), metronidazole (3/17), ceftazidime (1/17), clindamycin (1/17), doxycycline (2/17), and piperacillin-tazobactam (2/17). Several patients received more than one agent. Antibiotics recorded in the 6 months prior to presentation included amoxicillin-clavulanate (3/3), chloramphenicol (1/3), enrofloxacin (1/3), doxycycline (1/3), and metronidazole (2/3). Due to the retrospective nature of this study, complete antibiotic history was not available for all patients.

Hyperglycemia was found to have a significant association with survival. Animals with high glucose levels had better survival outcomes compared to those with normoglycemia and hypoglycemia in this patient population (*p =* 0.0099). The results of these categorical comparisons, including leukocyte count, glucose, bilirubin, blood pressure, presence of toxic change, and antibiotic use, are summarized in Table 1. Descriptive statistics for leukocyte counts, glucose levels, and total bilirubin by survival group, and subdivided by species, are presented in Table 2.

Of the 16 patients, 6 had cultures submitted after PBS-detected bacteremia was noted. The patients with blood cultures submitted included 3 dogs and 3 cats. Three of the submitted cultures yielded bacterial growth with species and susceptibility profiles. Five of the six had received antibiotics prior to culture collection. Among the 3 patients with positive cultures, all of which were non-survivors, two met the study’s outlined sepsis criteria (Section 2.2). Two were dogs and one was a cat. All had leukocytosis (range: 17.6–26.83 K/μL; mean: 20.6 K/μL), band neutrophils, toxic change, and hyperbilirubinemia (range: 0.7–8.7 mg/dL; mean: 3.7 mg/dL). Glucose levels were within reference intervals (range: 99–106 mg/dL; mean: 102.6 mg/dL). In one dog, the blood culture was positive for *Group G beta-hemolytic Streptococcus*, suspected to be due to a septic joint. In a second dog, the blood culture was positive for *Leclercia adecarboxylata*, associated with a dialysis catheter. The cat had a blood culture that was positive for *Streptococcus dysgalactiae* subsp. *Equisimilis*, suspected to be due to urosepsis. In all three cases, the cultured organism matched the bacterial morphology noted on initial PBS review.

## 4 Discussion

Peripheral blood smears can be useful in detecting bacteremia in sick small animal patients and may provide diagnostic and prognostic information that can hasten the onset of treatment (15, 16). This study aimed to evaluate the significance of detecting bacteremia by PBS and its relationship with survival to discharge in a sick small animal population. No cases of bacteremia detected were found to be inconsequential in this patient population given that all were clinically ill at presentation. The findings suggest that bacteremia identified on PBS in clinically ill small animal patients is associated with a poor prognosis for survival.

All patients included in this study were clinically ill at time of presentation and PBS evaluation. However, asymptomatic animals were not intentionally excluded. All cases where bacteremia was identified on PBS during a retrospective review of electronic medical records were included. All animals identified had presented through the ED for various clinical signs. No instances of PBS-detected bacteremia were found for routine wellness visits or in other non-emergent settings. This supports the clinical impression that the presence of bacteremia found on PBS is a rare, but significant finding. However, it should be noted that the pathologists at the included institutions do not provide blood smear evaluations for all peripheral blood samples submitted to the laboratory. Instead, the pathologist review is triggered by a technologist alert, or a CBC abnormality. Because of this, it is possible that the results could be biased toward sick patients, decreasing the overall detected incidence of bacteremia identified on PBS in the population, and potentially overlooking bacteremia seen on blood smears of healthy pets.

Hyperglycemia was positively associated with survival. This finding is consistent with existing literature suggesting that hyperglycemia in septic or critically ill patients may not always be detrimental, and may reflect a metabolic response to stress (17, 18). In human and veterinary medicine, transient hyperglycemia has been observed in association with systemic inflammation, trauma, congestive heart failure, and sepsis, where it may play a protective role by supporting increased energy demands of immune cells (17, 19). Conversely, persistent or severe hyperglycemia has been associated with poorer outcomes in prolonged critical illness (19–21). The finding that hyperglycemia was associated with better outcomes in this cohort adds to the ongoing discussion about stress hyperglycemia in acute illness (17). All survivors in this study were cats, a species known to exhibit stress hyperglycemia due to adrenergic stimulation during acute illness or hospitalization (12, 22). It is possible that transient stress hyperglycemia in these patients served as a physiologic response to support increased energy demands, rather than indicating a primary derangement in glucose metabolism. In this context, hyperglycemia may be an adaptive response rather than a negative prognostic indicator.

Subgroup analysis was of low value given the small number of patients and thus was not reported. The lack of significant survival differences related to toxic change or band neutrophils is consistent with earlier research that suggests these indicators are not necessarily predictive of poor outcomes in small animals (23). However, one equine study did find the presence of band neutrophils and toxic change to be associated with decreased survival (24). To avoid overinterpretation of these findings, no single clinical or hematologic parameter should be relied upon to determine prognosis. Rather, a multifactorial approach is beneficial when evaluating animals with PBS-detected bacteremia, particularly given the small sample size and retrospective nature of this study.

Given the rarity of PBS-detected bacteremia, all eligible cases across species were included to capture the span of this clinical finding. Though bacteremia identified on PBS is a reported finding in reptile species, it remains rare in both domesticated bearded dragon and small mammal species (25). To date, literature related to bacteremia on PBS in rabbits has been limited to experimental models of sepsis and hematological studies (26). Case reports in domestic rabbits have noted bacteremia diagnosed by culture or associated histopathology, with minimal documentation of direct visualization on PBS. This highlights the novelty of PBS-detected bacteremia in rabbits. While no prognostic conclusions cannot be drawn from a single case, this finding may warrant further consideration in future studies if additional cases are identified.

In addition to being a poor prognostic indicator for survival, PBS-detected bacteremia may also serve as an early indicator of sepsis. However, interpreting bacteremia on PBS as an indicator of sepsis is complicated by the current lack of consensus on how sepsis is clinically identified in veterinary medicine (4). Additionally, due to the retrospective nature of this study, it was not possible to uniformly apply SIRS criteria, definitively identify sources of infection, or calculate APPLE_full_ or SOFA scores for all patients (4, 14). Given the retrospective design, small sample size, and limitations in sepsis classification, the primary objective of this study was to directly evaluate the association between PBS-detected bacteremia and survival to discharge. A future prospective study that more specifically explores the relationship between PBS-detected bacteremia and the incidence of sepsis would be a valuable contribution to the current body of literature.

This study had several limitations, primarily related to its small sample size, lack of a comparator group, and its retrospective design. While exploratory patterns were observed, the small sample size limits definitive conclusions regarding associations between specific clinical parameters and survival outcomes. Antibiotic selection was not standardized, which may limit the interpretation of its effect on survival. The study lacked a comparator group of patients without bacteremia on PBS, and as such, conclusions are limited to associations within this cohort. Furthermore, the variation in diagnostic and treatment approaches between patients could have influenced the outcomes. Another limitation of this study is the potential influence of PBS-detected bacteremia on euthanasia decisions. Though none of the cases were noted to be euthanized solely based on PBS findings from the records, it is possible that visualization of bacteria on a blood smear could have contributed to clinician perception of a poor prognosis. Because of this, we cannot definitively determine the extent to which PBS findings may have biased outcomes as retrospective records did not uniformly document rationale for euthanasia. Future studies would need a larger, multicenter cohort to strengthen the statistical power of these findings. As we get closer to defining the parameters involved in the diagnosis of sepsis, standardization of diagnostic and treatment protocols could provide clearer guidance for clinicians managing patients with bacteremia.

In conclusion, bacteremia identified on PBS in clinically ill small animal patients is a rare, but meaningful diagnostic finding that is associated with poor prognosis for survival to discharge. The presence of PBS-detected bacteremia supports the importance of timely diagnostics and consideration of early intervention, though prospective studies are needed to determine its impact on treatment decisions (9, 25). Though not all hospitals have an in-house clinical pathology laboratory, PBS can still function as a useful bedside tool for emergency clinicians who are awaiting the results of more definitive testing such as blood cultures and have a high clinical suspicion for infection. Incorporating PBS evaluation into emergency settings may facilitate earlier recognition of serious illness and support timely therapeutic interventions.

## Data Availability

The original contributions presented in the study are included in the article/Supplementary material, further inquiries can be directed to the corresponding author.
