# Supplementary material for: Bacteremia detected on peripheral blood smear in small animal patients presenting to the Emergency Department and its association with prognosis to discharge
Source: Front Vet Sci. 2025 Jun 4;12:1550732. doi: 10.3389/fvets.2025.1550732 (PMC12173895; doi:10.3389/fvets.2025.1550732)
Supplement: Supplementary file 1 [file Image_1.pdf]

Supplementary Material

1.1 Supplementary Figure

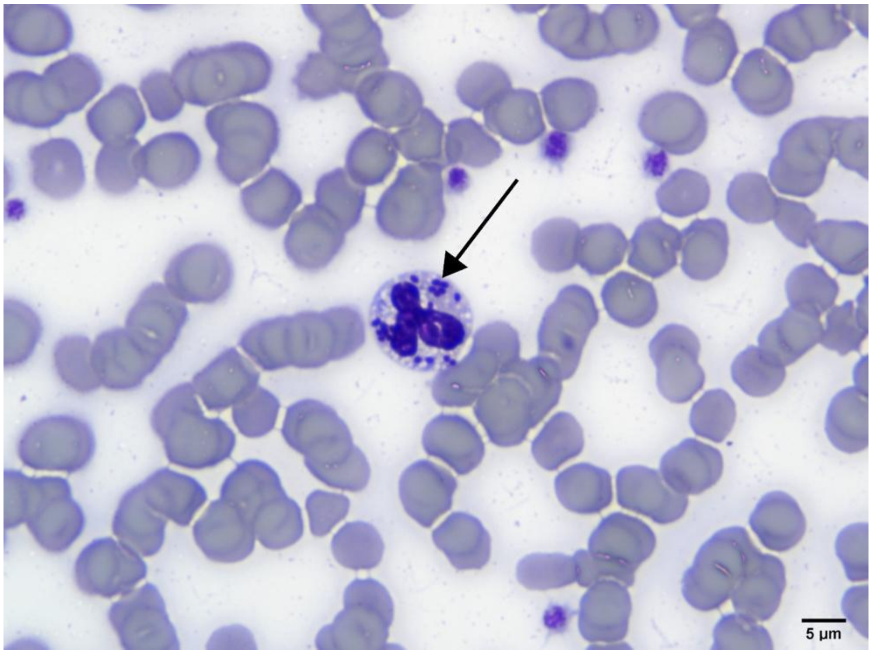

Figure 1. Peripheral blood smear from a bacteremic cat. A neutrophil (indicated by the black arrow) demonstrates marked toxic change and contains multiple phagocytized bacterial cocci. This finding was noted on a Wright-Giemsa stained smear evaluated at 1000× magnification. Image courtesy of Dr. Patty Ewing, DVM, MS, DACVP.
